# Letter from Dr. Edmonds, Buffalo

**Published:** 1859-12

**Authors:** J. J. Edmonds

**Affiliations:** Buffalo


PROF. AUSTIN FLINT, JR.:

*Dear Sir.*—In the "*American Medical Monthly*," for October, 1859, are some remarks by Dr. O. C. Gibbs, of Frewsburg, N. Y., in relation to my report of a "Case of gun-shot wound through the abdomen," published in the August number of this Journal. I do not complain because his opinions differ from my own, for I conceive that he has a perfect right to believe or disbelieve whatever he pleases; but Dr. Gibbs has, as I shall presently demonstrate, misquoted my language, and made me responsible, unjustly, for an error in grammar which a school-boy would blush to commit. He has also put my words in italics, without explanation, all of which he had no right to do; and therefore I can do no less than call him to account.

I proceed to quote from his remarks. He says: "On the eighth day of the treatment with six grs. of morphine daily, the bowels moved spontaneously '*and quite natural.*' Had the intestines been riddled with the ball, we must be permitted to think that the evacuations would not have been *quite natural*, and the result, we may suppose, might have been different." (See American Medical Monthly for October, 1859, p. 284.)

Now, the paragraph in my report, from which the doctor pretended to quote the above, reads in this wise: "March 8th—Patient gaining; bowels have moved freely, and spontaneously, three or four times; pulse 88 per minute, and quite natural," &c. (See this Journal for August, 1859, p. 150.) The reader will mark the difference. The words "quite natural" refer to the *pulse*, and are therefore in their proper place, which would not be the case if they were placed to qualify the verb *moved*. A few words in relation to my former article, and I have done. It strikes me that the facts noted above, on which Dr. Gibbs bases his opinion, that the intestines had not been "riddled" (riddled with one ball!) furnish pretty strong evidence to the contrary. It strikes me that the free and spontaneous evacuation of the bowels, four times during the twenty-four hours, while the patient was taking the large amount of morphine mentioned, was anything but "natural;" and then the direction of the wound, as indicated conclusively by the probe, was directly through the abdomen. Then, again, the ball was lodged in the abdominal muscles, precisely opposite to the external wound in the back. These items, taken in connection with the fact that severe peritonitis was present, justify me, I think, in repeating my words, used in the former article: that "there could be no mistake, or room for doubt." Nor can I see that the case, viewed in this light, affords any room for wonder or surprise, as it merely offers additional evidence of the wonderful efficacy of opium, or its alkaloids, in the treatment of peritonitis.

Respectfully, &c.,

J. J. Edmonds.

Buffalo, *Nov.* 13*th*, 1859.

